# Outcomes of Blood Transfusions in Patients Undergoing Mechanical Thrombectomy for Acute Ischemic Stroke: A Population-Based Cross-Sectional Study of 47,835 Patients

**DOI:** 10.3390/brainsci15040386

**Published:** 2025-04-08

**Authors:** Ankita Jain, Eseiwi Aifuwa, Raphael Bienenstock, Shayna Kar, Eris Spirollari, Ariel Sacknovitz, Elad Mashiach, Feliks Koyfman, Ji Chong, Chaitanya Medicherla, Chirag D. Gandhi, Fawaz Al-Mufti

**Affiliations:** 1 School of Medicine, New York Medical College, Valhalla, NY 10595, USA; ajain9@student.nymc.edu (A.J.); eaifuwa2@student.nymc.edu (E.A.); rbienens2@student.nymc.edu (R.B.); espiroll@student.nymc.edu (E.S.); asacknov2@student.nymc.edu (A.S.); chirag.gandhi@wmchealth.org (C.D.G.); 2 Department of Neurosurgery, Westchester Medical Center, Valhalla, NY 10595, USA; elad.mashiach@wmchealth.org; 3 Department of Neurology, Westchester Medical Center, Valhalla, NY 10595, USA; shayna.kar@vanderbilt.edu (S.K.); feliks.koyfman@wmchealth.org (F.K.); ji.chong@wmchealth.org (J.C.); chaitanya.medicherla@wmchealth.org (C.M.)

**Keywords:** endovascular thrombectomy, blood transfusions, acute ischemic stroke, large vessel occlusion, in-hospital mortality

## Abstract

**Background/Objectives**: Despite advances, large vessel occlusion strokes (LVO) remain associated with significant morbidity. Recent studies have suggested that blood transfusions may help manage critically ill LVO patients. We sought to evaluate the patient characteristics, complications, and clinical outcomes associated with blood transfusions in acute ischemic stroke (AIS) patients undergoing endovascular thrombectomy. **Methods**: A query of the 2016–2019 National Inpatient Sample was conducted to identify AIS patients who underwent endovascular thrombectomy, using International Classification of Disease 10th Revision diagnostic codes. Demographic, clinical characteristics, severity of presentation, complications, and outcomes were analyzed. Multivariate binary logistic regression was used to assess complications, length of stay (LOS), discharge disposition, and inpatient mortality. **Results**: A total of 47,835 AIS patients undergoing endovascular thrombectomy were identified. Of these patients, 1215 (2.5%) received blood transfusions. After controlling for age, gender, National Institutes of Health Stroke Scale scores, Elixhauser Comorbidity Index, and location of stroke, blood transfusions were significant positive predictors for higher rates of inpatient death (OR: 1.96; 95% CI: 1.681, 2.286; *p* < 0.001), lower rates of routine discharge (OR: 0.425; 95% CI: 0.342, 0.527; *p* < 0.001), and prolonged LOS (OR: 2.928; 95% CI: 2.572, 3.333; *p* < 0.001). **Conclusions**: Blood transfusions in AIS patients receiving endovascular thrombectomy are associated with elevated complication rates, extended hospital stays, and increased mortality, even after for controlling for predictors of poor outcome. Understanding the broader effects of blood transfusions in AIS patients is essential to ensure that the balance between potential benefits and risks upholds best care practice for all patients.

## 1. Introduction

Large vessel occlusion (LVO) resulting in acute ischemic stroke (AIS) is a critical and prevalent condition, significantly contributing to the overall burden of stroke in the United States. In 2019, strokes accounted for 5.26% of all deaths in the United States [1]. LVO strokes comprise up to 38% of AIS, resulting in larger infarct size, increased presenting severity, and poorer long-term outcomes, compared to non-LVO AIS [2,3]. Common causes of LVO-induced AIS include large artery atherosclerosis and cardioembolism [4,5].

With strong potential for significant morbidity and mortality in the setting of LVO stroke, management of this condition focuses on swift restoration of blood flow to minimize ischemic time and improve outcomes. Initial treatment typically involves intravenous thrombolysis with tissue plasminogen activator (tPA) or tenecteplase [6]. However, the effectiveness of these thrombolytics in LVO stroke is limited, with successful recanalization rates ranging from 8% to 32% [7]. Mechanical thrombectomy, otherwise known as endovascular thrombectomy (EVT), offers significant benefits in the treatment of LVO and can be performed up to 24 h from the last known well in select patients [8]. Combining timely thrombolytic administration with EVT represents the current standard of care, enhancing recovery and mitigating the long-term impact of LVO strokes.

Recent literature on large-vessel AIS has suggested that blood transfusions may play a role in managing AIS. Concomitant anemia is found in 40% of AIS patients, and AIS patients presenting with anemia have a 15.2% increase in risk of mortality compared to those without anemia [9]. As a result, ensuring blood homeostasis through transfusions may be a valuable strategy to maintain cerebral perfusion and prevent progression and complications in patients experiencing AIS. Furthermore, previous animal studies demonstrated that transfusing fresh, nonischemic blood markedly reduced infarct size and neurologic deficits, and provided platelets that helped maintain microvascular integrity and decreased the risk of adverse tPA-associated hemorrhage [10,11]. However, there is limited literature addressing the direct impact of blood transfusions on post-stroke morbidity and mortality in human subjects. This population-based, cross-sectional study explores patient characteristics, complications, and outcomes of blood transfusions in LVO-induced AIS.

## 2. Materials and Methods

### 2.1. Data Source and Patient Selection

The National Inpatient Sample (NIS) database, created for the Healthcare Cost and Utilization Project by the Agency of Healthcare Research and Quality, was utilized for this study. The NIS is the largest, all-payer inpatient database in the United States. It is a stratified discharge database encompassing data from 4550 hospitals across 48 states, representing 20% of all inpatient admissions and over 7 million hospital stays annually [12]. NIS was queried from 2016–2019 for patients with AIS using International Classification of Disease 10th Revision (ICD-10) diagnostic codes. Patients were further stratified to those undergoing mechanical thrombectomy. Since NIS does not contain any identifying patient information, approval from an institutional review board was not required for this study.

### 2.2. Data Characteristics and Outcome Measures

Demographics and clinical characteristics—including patient age, gender, ethnicity, comorbidities, risk factors, and insurance status—were analyzed for AIS patients who received blood transfusions during their inpatient admission and those who did not. Severity of presentation, complications during admission, and other sequelae, including deep vein thrombosis (DVT), pulmonary embolism (PE), acute kidney injury (AKI), aphasia, hemiplegia, herniation, coma, seizure, cerebral edema, requirement of tracheostomy, and requirement of mechanical ventilation, were assessed. The primary outcome measures examined were prolonged length of stay (≥6 days; LOS), discharge disposition, and inpatient death.

### 2.3. Statistical Analysis

Categorical variables were compared using Pearson’s Chi-square test. A multivariate binary logistic regression was conducted to assess the outcomes of complications, prolonged LOS, discharge disposition, and inpatient mortality. The regression analysis controlled for age, gender, National Institutes of Health Stroke Scale (NIHSS) scores, Elixhauser Comorbidity Index, and location of stroke. All statistical analyses were performed using Statistical Product and Service Solutions (V. 28; IBM, Armonk, NY, USA).

## 3. Results

A total of 47,835 AIS patients who underwent EVT were identified. Of these patients, 1215 (2.5%) received blood transfusions. Of the AIS patients who received blood transfusions, 66.7% were female (OR: 1.932; 95% CI: 1.712, 2.179; *p* < 0.001), 66.3% were Caucasian (OR: 0.957; 95% CI: 0.848, 1.079; *p* = 0.477), and 53.1% were in the lower 50th percentile median income (OR: 1.023; 95% CI: 0.913, 1.147; *p* = 0.705) (Table 1).

### 3.1. Complications

Patients who received blood transfusions had higher rates of DVT (OR: 1.635; 95% CI: 1.279, 2.092; *p* < 0.001), AKI (OR: 2.519; 95% CI: 2.218, 2.862; *p* < 0.001), cerebral edema (OR: 1.538; 95% CI 1.353, 1.748; *p* < 0.001), cerebral herniation (OR: 2.381; 95% CI: 2.028, 2.796; *p* < 0.001), requirement of tracheostomy (OR: 5.13; 95% CI: 4.205, 6.259; *p* < 0.001), and requirement of mechanical ventilation (OR: 2.648; 95% CI: 2.326, 3.014; *p* < 0.001), compared to patients who did not receive blood transfusions. The rate of coma was not significantly different between patients who received blood transfusions and those who did not (OR: 1.041; 95% CI: 0.918, 1.18; *p* = 0.539). Furthermore, there were no significant differences in the rates of aphasia (OR: 0.972; 95% CI: 0.867, 1.09; *p* = 0.642), PE (OR: 1.276; 95% CI: 0.853, 1.909; *p* = 0.249) or hemiplegia (OR: 1.16; 95% CI: 0.993, 1.354; *p* = 0.064) between the two groups. However, the rate of seizure was lower in AIS patients who received blood transfusions (OR: 0.64; 95% CI: 0.466, 0.879; *p* = 0.006) compared to patients who did not (Table 2).

### 3.2. Outcomes

AIS patients with EVT receiving blood transfusions were more likely to experience prolonged LOS (OR: 3.389; 95% CI: 2.989, 3.842; *p* < 0.001), transfer to a skilled nursing facility (OR: 1.163; 95% CI: 1.036, 1.306; *p* = 0.01), and inpatient death (OR: 1.563; 95% CI: 1.492, 1.638; *p* < 0.001) compared to AIS patients who did not receive blood transfusions (Table 3). Furthermore, rates of routine discharge (OR: 0.342; 95% CI: 0.279, 0.418; *p* < 0.001) were decreased in those receiving blood transfusions. There was no significant difference in the rates of home health care (OR: 0.991; 95% CI: 0.816, 1.203; *p* = 0.961) or transferring patients to a short-term hospital (OR: 1.024; 95% CI: 0.744, 1.409; *p* = 0.934). After multivariate logistic regression analysis, blood transfusions in AIS patients were independent positive predictors for higher rates of inpatient death (OR: 1.96; 95% CI: 1.681, 2.286; *p* < 0.001), lower rates of routine discharge (OR: 0.425; 95% CI: 0.342, 0.527; *p* < 0.001), and prolonged LOS (OR: 2.928; 95% CI: 2.572, 3.333; *p* < 0.001) (Table 4).

## 4. Discussion

This study is the first of its kind to analyze the clinical outcomes of blood transfusions in patients undergoing EVT for LVO stroke. We found that patients who received blood transfusions were more likely to be female (OR: 1.932; 95% CI: 1.712, 2.179; *p* < 0.001) and have a higher Elixhauser Comorbidity Index (6.2646 ± 2.00033; *p* < 0.001) compared to patients who did not receive blood transfusion. Notably, after controlling for age, gender, NIHSS scores, Elixhauser Comorbidity Index, and location of stroke, blood transfusions were positive predictors for prolonged LOS (OR: 2.928; 95% CI: 2.572, 3.333; *p* < 0.001) and higher rates of inpatient death (OR: 1.96; 95% CI: 1.681, 2.286; *p* < 0.001).

We investigated LOS as one of several complications examined in AIS patients receiving EVT and blood transfusions. The current findings align with those of a previous retrospective cohort study that compared the outcomes of patients with various acute brain injury subtypes who received blood transfusions with those who did not [13]. The study analyzed outcomes in 1142 patients with AIS and found that, among the 67 patients who received blood transfusions, there was a significant increase in LOS compared to patients who did not receive blood transfusions. An additional retrospective study demonstrated that transfused patients at similar hemoglobin levels of 7–9 g/dL had longer ICU (8 vs. 3.5 days; *p* < 0.001) and hospital stays (20 vs. 11 days; *p* < 0.001) [14]. A separate retrospective cohort study found that patients who were transfused a single unit of red blood cells were more likely to have an increased LOS and had 1.42 times higher odds of 28-day readmission (95% CI: 1.20–1.69; *p* < 0.001) [15]. The results of the current study corroborate and expand on previous findings that blood transfusions are associated with increased LOS in AIS patients. This study emphasizes the need for greater attention to various markers and conditions in these patients, so as to proactively optimize their care and potentially limit LOS and other complications.

This study found an increased mortality rate for AIS patients who were treated with EVT and blood transfusions. A recent worldwide audit of ICU and hospital outcomes in critically ill patients found that hospital mortality rates were higher in transfused patients compared to non-transfused patients (30.0% vs. 19.6%; *p* < 0.001) [16]. It was also demonstrated that the mortality rate increased with increasing numbers of transfused units [16]. The PATCH trial observed the relationship between platelet transfusion and mortality in patients who experienced acute stroke due to spontaneous cerebral hemorrhage. This trial found that the odds of dependence or death at three months were higher in the platelet transfusion group than in the standard care group (OR: 2.05; 95% CI: 1.18, 3.56; *p* = 0.0114) [17]. This contradicts prior animal studies that demonstrated that blood and platelet transfusions helped protect neuro-vasculature and decreased the risk tPA-associated hemorrhage [10,11]. In addition, a previous observational retrospective study found that patients who received transfusions in cardiac surgery also suffered from increased mortality. The transfusion of unidentified pro-inflammatory cytokines in the blood samples could exacerbate tissue ischemia [18]. Furthermore, previous studies have demonstrated that blood transfusions are associated with electrolyte disturbances, volume overload, and transfusion reactions [19,20,21]. These outcomes emphasize the importance of careful evaluation of the necessity and timing of blood transfusions in AIS patients to effectively balance the benefits and risks of transfusion.

It is important to recognize that the necessity for blood transfusions in AIS patients undergoing EVT is often associated with a generally worse condition of the patients and a higher likelihood of serious complications. As such, the correlative relationship between blood transfusions and worse clinical outcomes in these patients needs to be interpreted with caution. The need for blood transfusions in AIS patients receiving EVT may indicate more severe underlying conditions in the patients, as opposed to direct contribution to poor clinical outcomes.

### Limitations

Several limitations are inherent to the utilization of the NIS database. The use of ICD-10 codes to identify patient cohorts and outcomes can introduce variability and potential misclassification, due to inconsistent coding practices across hospitals. However, this study sought to mitigate further heterogeneity and errors by focusing the analysis on 2016–2019, thus avoiding the shift from ICD-9 to ICD-10 coding that occurred in 2015. Additionally, the NIS lacks detailed clinical data—such as specifics of transfusion practices, recanalization measurement, procedure-related hemorrhagic complications, etc.—which may affect the interpretation of outcomes. For example, estimated blood loss during each EVT procedure is not reported in the NIS database, which could impact the overall necessity of a blood transfusion. Furthermore, the NIS does not provide long-term follow-up data, restricting the analysis to in-hospital outcomes and preventing assessment of long-term morbidity and mortality. Future prospective studies are needed to validate the findings of this study and to provide a more comprehensive understanding of the impact of blood transfusions on AIS patients undergoing EVT.

## 5. Conclusions

This population-based, retrospective study found that blood transfusions in the setting of AIS patients undergoing EVT are associated with higher rates of complications, prolonged hospital stays, and increased inpatient mortality. These findings highlight the importance of weighing the potential advantages and disadvantages of blood transfusion when managing AIS patients undergoing EVT. Ultimately, these findings warrant a careful reevaluation of blood transfusion practices in the management of AIS as understanding the critical interplay between intervention and outcome may inform the difference between recovery and fatality.

## Figures and Tables

**Table 1:** Baseline demographics and comorbidities of patients undergoing EVT for AIS stratified by blood transfusion status.

Variables	Total Cohort (47,835)	Non-Blood Transfusion (46,620, 97.5%)	Blood Transfusion (1215, 2.5%)	Blood Transfusion	
				OR (CI 95%)	*p*-Value
Demographics					
Female	24,525 (51.3%)	23,715 (50.9%)	810 (66.7%)	1.932 (1.712, 2.179)	<0.001
Lower 50th Percentile Median Income	25,125 (52.5%)	24,480 (52.5%)	645 (53.1%)	1.023 (0.913, 1.147)	0.705
Private Insurance	10,920 (22.8%)	10,630 (22.8%)	290 (23.9%)	1.061 (0.929, 1.213)	0.387
Medicare Insurance	29,285 (61.2%)	28,570 (61.3%)	715 (58.8%)	0.903 (0.805, 1.014)	0.089
Medicaid Insurance	4610 (9.6%)	4460 (9.6%)	150 (12.3%)	1.331 (1.119, 1.584)	0.001
White Race	32,150 (67.2%)	31,345 (67.2%)	805 (66.3%)	0.957 (0.848, 1.079)	0.477
Comorbidities					
Diabetes Mellitus	13,005 (27.2%)	12,695 (27.2%)	310 (25.5%)	0.915 (0.803, 1.043)	0.191
Hypertension	25,000 (52.3%)	24,500 (52.6%)	500 (41.2%)	0.631 (0.562, 0.709)	<0.001
Hyperlipidemia	23,440 (49%)	22,970 (49.3%)	470 (38.7%)	0.65 (0.578, 0.73)	<0.001
Obesity	4755 (9.9%)	4660 (10%)	95 (7.8%)	0.764 (0.618, 0.944)	0.013
Chronic Obstructive Pulmonary Disease	5565 (11.6%)	5415 (11.6%)	150 (12.3%)	1.072 (0.901, 1.274)	0.441
Congestive Heart Failure	2330 (4.9%)	2255 (4.8%)	75 (6.2%)	1.294 (1.021, 1.641)	0.036
Smoker Status	9140 (19.1%)	8965 (19.2%)	175 (14.4%)	0.707 (0.601, 0.831)	<0.001
Alcohol Abuse Disorder	2670 (5.6%)	2590 (5.6%)	80 (6.6%)	1.198 (0.952, 1.508)	0.128
Chronic Anticoagulant/ Antiplatelet Use	8920 (18.6%)	8725 (18.7%)	195 (16%)	0.83 (0.711, 0.969)	0.019
Intravascular Thrombolytics	11,040 (23.1%)	10,810 (23.2%)	230 (18.9%)	0.774 (0.669–0.894)	0.001
Patients with Stroke Location Identified					
Posterior Circulation Stroke	43,205 (90.3%)	42,100 (90.3%)	1105 (90.9%)	1.079 (0.884–1.315)	0.462
Anterior Circulation Stroke	3160 (6.6%)	3080 (6.6%)	80 (6.6%)	0.996 (0.792–1.254)	1
Variables	Total Cohort Mean (St. Dv.)	Non-Blood Transfusion Mean (St. Dv.)	Blood Transfusion Mean (St. Dv.)	*p*-Value	
Age	68.86 (±14.839)	68.85 (±14.841)	69.35 (±14.789)	0.240	
Elixhauser Comorbidity Index	5.2785 (±1.88642)	5.2554 (±1.87780)	6.2646 (±2.00033)	<0.001	
NIHSS	15.8003 (±7.60538)	15.7706 (±7.61439)	16.9383 (±7.16195)	<0.001	

NIHSS = National Institutes of Health Stroke Scale Score.

**Table 2:** Hospital course of patients undergoing EVT for AIS stratified by blood transfusion status.

Variables	Total Cohort (47,835)	Non-Blood Transfusion (46,620, 97.5%)	Blood Transfusion (1215, 2.5%)	Blood Transfusion	
				OR (CI 95%)	*p*-Value
DVT	1750 (3.7%)	1680 (3.6%)	70 (5.8%)	1.635 (1.279, 2.092)	<0.001
PE	780 (1.6%)	755 (1.6%)	25 (2.1%)	1.276 (0.853, 1.909)	0.249
AKI	6685 (14%)	6340 (13.6%)	345 (28.4%)	2.519 (2.218, 2.862)	<0.001
Tracheostomy	1095 (2.3%)	975 (2.1%)	120 (9.9%)	5.13 (4.205, 6.259)	<0.001
Aphasia	24,935 (52.1%)	24,310 (52.1%)	625 (51.4%)	0.972 (0.867, 1.09)	0.642
Hemiplegia	39,180 (81.9%)	38,160 (81.9%)	1020 (84%)	1.16 (0.993, 1.354)	0.064
Herniation	3455 (7.2%)	3270 (7%)	185 (15.2%)	2.381 (2.028, 2.796)	<0.001
Coma	13,400 (28%)	13,050 (28%)	350 (28.8%)	1.041 (0.918, 1.18)	0.539
Mechanical Ventilation	6085 (12.7%)	5755 (12.3%)	330 (27.2%)	2.648 (2.326, 3.014)	<0.001
Seizure	2395 (5%)	2355 (5.1%)	40 (3.3%)	0.64 (0.466, 0.879)	0.006
Cerebral Edema	9585 (20%)	9250 (19.8%)	335 (27.6%)	1.538 (1.353, 1.748)	<0.001

DVT = Deep Vein Thrombosis; PE = Pulmonary Embolism; AKI = Acute Kidney Injury.

**Table 3:** Outcomes of patients undergoing EVT for AIS stratified by blood transfusion status.

Variables	Total Cohort (47,835)	Non-Blood Transfusion (46,620, 97.5%)		Blood Transfusion (1215, 2.5%)		Blood Transfusion	
						OR (CI 95%)	*p*-Value
Prolonged Length of Stay > 6 Days	20,525 (42.9%)	19,660 (42.2%)		865 (71.2%)		3.389 (2.989–3.842)	<0.001
Routine Discharge	10,210 (21.3%)	10,105 (21.7%)		105 (8.6%)		0.342 (0.279–0.418)	<0.001
Home Health Care	4565 (9.5%)	4450 (9.5%)		115 (9.5%)		0.991 (0.816–1.203)	0.961
Transfer to Short Term Hospital	1540 (3.2%)	1500 (3.2%)		40 (3.3%)		1.024 (0.744–1.409)	0.934
Transfer to Skilled Nursing Facility	26,220 (54.8%)	25,510 (54.7%)		710 (58.4%)		1.163 (1.036–1.306)	0.01
Leave Against Medical Advice	130 (0.3%)	130 (0.3%)		0 (0%)		0.997 (0.997–0.998)	0.084
Inpatient Death	40,745 (22%)	38,055 (21.6%)		2690 (30.1%)		1.563 (1.492–1.638)	<0.001
Variables	Total Cohort Mean (St. Dv.)		Non-Blood Transfusion Mean (St. Dv.)		Blood Transfusion Mean (St. Dv.)		*p*-Value
Length of Stay	8.01 (±9.016)		7.82 (±14.098)		15.19 (±18.572)		<0.001
Total Charges	193,262.15 (±158,504.767)		190,477.39 (±153,188.438)		302,633.31 (±279,289.332)		<0.001

**Table 4:** Independent predictors of outcomes in patients undergoing mechanical thrombectomy for acute ischemic stroke.

Variables *	Inpatient Death		Routine Discharge		Prolonged Length of Stay > 6 Days	
	OR (CI 95%)	*p*-Value	OR (CI 95%)	*p*-Value	OR (CI 95%)	*p*-Value
Blood Transfusion	1.96 (1.68–2.286)	<0.001	0.425 (0.342–0.527)	<0.001	2.928 (2.572–3.333)	<0.001

* Multivariate logistic regression controlling for age, gender, NIHSS, Elixhauser Comorbidity Index, and location of stroke.

## Data Availability

This study data can be requested from the corresponding author after completing the required procedures outlined by the Healthcare Cost and Utilization Project.

## Abbreviations

The following abbreviations are used in this manuscript:

LVO	Large Vessel Occlusion
AIS	Acute Ischemic Stroke
tPA	Tissue Plasminogen Activator
EVT	Endovascular Thrombectomy
NIS	National Inpatient Sample
ICD	International Classification of Disease
DVT	Deep Vein Thrombosis
PE	Pulmonary Embolism
AKI	Acute Kidney Injury
LOS	Length of Stay
NIHSS	National Institutes of Health Stroke Scale
